# Astragaloside IV attenuates sunitinib-associated cardiotoxicity by inhibiting COUP-TFII

**DOI:** 10.1016/j.heliyon.2024.e24779

**Published:** 2024-01-18

**Authors:** Wanting Qin, Shaoling Li, Ziji Cheng, Wenlong Xue, Mingyue Tian, Fangfang Mou, Haidong Guo, Shuijin Shao, Baonian Liu

**Affiliations:** a Department of Anatomy, College of Chinese Integrative Medicine, Shanghai University of Traditional Chinese Medicine, Shanghai, 201203, China; b Department of Pathology, Shanghai Pulmonary Hospital, School of Medicine, Tongji University, Shanghai, 200433, China; c Department of Physiology and Pathophysiology, School of Basic Medical Sciences, Fudan University, Shanghai, 200032, China

**Keywords:** Astragaloside IV, Sunitinib-associated cardiotoxicity, COUP-TFII, Oxidative stress, Apoptosis

## Abstract

Sunitinib (SU) is widely used to treat solid tumors but it can be cardiotoxic and often leads to drug withdrawn or discontinuation. Astragaloside IV (ASIV) is the essential active component of the Chinese herb *Astragalus membranaceus* which shows potential cardioprotective effects. Herein, we investigated the effect of ASIV on SU-associated cardiotoxicity and its mechanisms. We showed that ASIV significantly ameliorated SU-induced myocardial injury in mice, as evidenced by an improvement in left ventricular ejection fraction (EF) and a decrease in blood pressure and serum concentration of myocardial injury markers. ASIV attenuated SU-induced myocardial inflammatory infiltration and fibrotic lesions. In addition, ASIV suppressed SU-induced myocardial oxidative stress and apoptosis both *in vitro* and *in vivo*. Furthermore, SU increased COUP-TFII expression both in mRNA and protein levels in mice myocardial tissue, primary neonatal rat cardiomyocytes (NRCMs) and H9c2 cell lines, and this effect was rescued by ASIV. Knockdown of COUP-TFII reduced the oxidative stress and apoptosis induced by SU in NRCMs and H9c2 cell lines. However, the overexpression of COUP-TFII blocked the protective effects of ASIV on SU-treated cardiomyocytes. Thus, our results demonstrated that ASIV ameliorated SU-indued cardiotoxicity by inhibiting COUP-TFII, suggesting that ASIV might be a potential therapeutic strategy for the prevention of SU-associated cardiotoxicity.

## Graphical abstract

**Figure:**
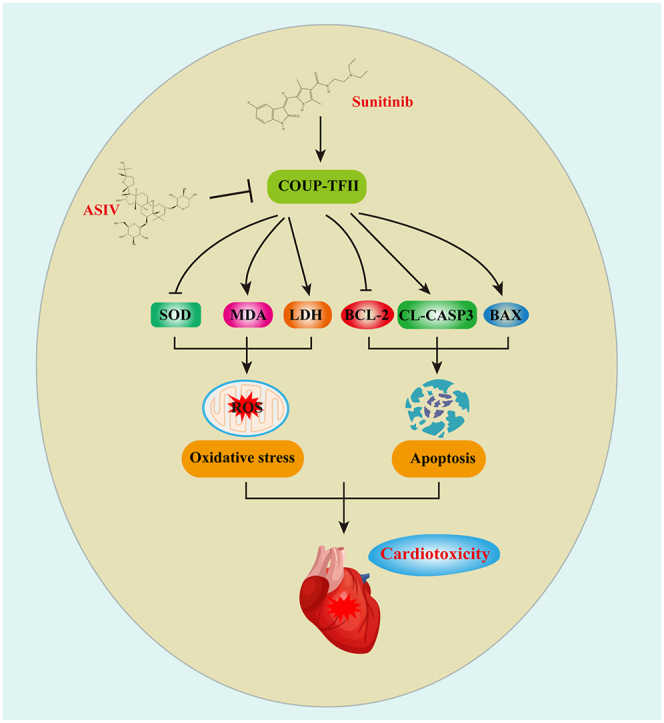

## 1 Introduction

Astragaloside IV (also known as 3-*O*-β-d-xylopyranosyl-6-*O*-β-D-*gluco*-pyranosyl-cycloastragenol, ASIV), the predominate phytochemical in traditional Chinese medicine *Astragalus membranaceus*, has been characterized as a potential therapeutic agent to tackle different cardiovascular disorders, such as ischemic heart disease, diabetic cardiomyopathy, heart failure and anthracycline-induced cardiotoxicity [[1], [2], [3]]. Moreover, ASIV confers its cardioprotective effects by improving myocardial energy metabolism; promoting angiogenesis; and inhibiting myocardial oxidative stress, inflammatory and apoptosis [1,2]. However, the precise mechanisms underlying SU-induced cardiotoxicity remain unclear.

Tyrosine kinase inhibitors (TKIs) have significantly improved cancer treatment but have been implicated in causing serious adverse cardiac events, which affects the prognosis of tumor patients and limits the clinical application of drugs [4,5]. Sunitinib (SU), an oral inhibitor of multi-targeted tyrosine kinase, is widely used to treat advanced renal cell carcinomas, gastrointestinal stromal tumors and advanced pancreatic neuroendocrine tumors. However, clinical practices and pivotal trials showed that treatment with SU was linked with serial adverse effects of cardiovascular, including QT prolongation, heart failure and hypertension, which are not curable in most patients even after the end of SU treatment [6,7]. Cancer patients whose treatment was terminated because of cardiotoxicity still deal with the second challenge of SU, leaving a huge cardiovascular burden for tumor patients.

Furthermore, SU induces mitochondrial injury, thereby impairing energy metabolism in cardiomyocytes and ultimately resulting in cardiac dysfunction [4,8]. Inhibition of AMP-activated protein kinase is considered an off-target effect of SU, which can reduce the capacity of cardiomyocytes to respond to energy stress [9,10]. Maladaptive autophagy promotes cardiomyocyte apoptosis and cardiotoxicity in SU [11]. Moreover, SU promotes cardiomyocyte oxidative stress and fibro-inflammatory myocardial processes, which may be the key reason for cardiotoxicity [11,12]. Although preliminary findings have been obtained, there is still a lack of effective treatment for SU-induced cardiotoxicity.

Nuclear receptors (NRs) belong to a superfamily of evolutionary conserved proteins which have been proven to be valuable pharmaceutical targets because they are key regulators of many diseases and have druggable ligand-binding sites [13]. Chicken ovalbumin upstream promoter-transcription factor II (COUP-TFII) is an orphan NR due to the lack of a known natural ligand [14]. Functionally, COUP-TFII regulates gene expression through directly binding to DNA elements or interacting with other transcription factors and cofactors [15]. Recently, COUP-TFII has been identified as an intriguing therapeutic target. COUP-TFII protein is highly expressed in the mesenchymal compartment during embryonic development, whereas, as development proceeds, its expression decreases considerably and it expressed at a low level in adult tissues [15,16]. However, accumulating evidence has shown that COUP-TFII expression is elevated under pathological conditions and COUP-TFII overexpression promotes progression of a spectrum of diseases, including cancer, heart failure and muscular dystrophy [[17], [18], [19], [20]]. Notably, suppression of COUP-TFII slows down disease progression, which makes it potential molecular target for disease treatment [21]. Nevertheless, whether COUP-TFII plays a role in SU-associated cardiotoxicity is still unknown. The present study was designed to explore the potential effects of ASIV on SU-associated cardiotoxicity and to explore the underlying mechanisms.

## 2 Materials and methods

### 2.1 Animal experiment and drug treatment

Male C57BL/6 mice (18–22 g) at 6 weeks were purchased from Shanghai University of Traditional Chinese Medicine's Experimental Animal Center. All mice were housed in light-and temperature-controlled barrier facilities (12/12 h light/dark cycle, 25 ± 2 °C) with ad libitum access to food and water. After 1 week of acclimatization, mice were randomly grouped as vehicle (control group), SU (40 mg/kg/day), SU (40 mg/kg/day) + ASIV (10 mg/kg/day), and SU (40 mg/kg/day) + ASIV (50 mg/kg/day) (n = 10 per group). SU (SU11248, Selleck, Houston, USA) and ASIV (A274907, Aladdin, Shanghai, China) were both dissolved in distilled water and given to mice via oral gavage at 1:00 p.m. for 28 consecutive days, and treatment with ASIV was started 1 h before the SU intragastric administration. The treatment dosages of SU and ASIV were selected according to previous studies [[22], [23], [24]].

### 2.2 Cell culture

H9c2 cells (the embryonic rat cardiomyocyte-derived cell line) were purchased from the Shanghai Institutes for Biological Sciences (Shanghai, China). H9c2 cells were cultured in Dulbecco's Modified Eagle Medium (DMEM) medium (11965118, Gibco, USA) supplemented with 10 % fetal bovine serum (FBS; 10100, Gibco, USA) and 1 % penicillin-streptomycin solution (V900929, Corning Cellgro, China) in a humidified atmosphere of 5 % CO_2_ at 37 °C. Primary neonatal rat cardiomyocytes (NRCMs) were isolated according to protocols described previously [25]. Briefly, hearts were isolated from one-day-old Sprague Dawley rats and washed in HBSS 3 times. Next, the atria and great vessels were trimmed off and tissues were cut into small pieces and digested 5 times by 0.1 % collagenase type II (17101015, Thermo Fisher, USA). Digestions were collected and cells were centrifuged. After differential attachment for 1.5 h to separate adherent cardiac fibroblasts, NRCMs were cultured in growth media containing DMEM, 10 % FBS, 1 % penicillin-streptomycin solution, and 100 μM bromodeoxyuridine (S7918, Selleck, Houston, USA).

### 2.3 Cell viability assay and drug treatment

H9c2 cells (1 × 10^4^ cells/well) were seeded onto 96-well plates and cultured in medium for 12 h before treatment. For assessment of SU-induced cell viability, H9c2 cell lines were treated with SU (0, 1, 2, 4, 6, 8, 10, 15, 20, or 25 μM) for 24 h. Furthermore, for assessment of the protective effect of ASIV on SU-induced cell viability, H9c2 cell lines were co-treated with SU (4 μM) and ASIV (10, 20, 30, 40, 50, 60, 80, 100, 150, or 200 μM) for 24 h. Subsequently, H9c2 cell viability was detected by using CCK8 assay (40203ES88, YEASEN, Shanghai, China) according to the standard procedure. Briefly, 10 μl of detection reagent and 90 μl DMEM were added to each culture plate's well. After 2 h of incubation, the absorbance was measured at 450 nm using a microplate reader (Synergy 2; BioTek, USA). The doses of SU (4 μM) and ASIV (50 μM) in cell experiments were determined by CCK8 assay. Thereafter, H9c2 cell lines or NRCMs were separated into four groups as follows: (i) Control, (ii) SU (4 μM), (iii) ASIV (50 μM), and (iv) SU (4 μM) + ASIV (50 μM). Cells were pretreated with ASIV for 1 h before co-treatment with SU for 24 h.

### 2.4 Echocardiography measurement

After 28 days of treatment, mice were anesthetized with 2 % isoflurane and evaluated using Vevo 2100 High-Resolution Imaging System (Visual Sonics Inc, ON, Canada). The following parameters were measured from M-Mode images of the left ventricle (LV) taken from parasternal long-axis view: left ventricular end-diastolic volume (LVEDV), left ventricular end-systolic volume (LVESV), left ventricular end-diastolic diameters (LVDd) and left ventricular end-systolic diameters (LVDs). Indices of LV systolic functions, including ejection fractions (EF%) and fractional shortening (FS%) were calculated as follows: EF (%) = (LVEDV - LVESV)/LVEDV × 100 and FS (%) = (LVDd - LVDs)/LVDd × 100. At least 3 consecutive cardiac cycles were measured and averaged for each mouse.

### 2.5 Blood pressure measurement

Blood pressure was measured by using a non-invasive tail-cuff system (BP2010A, Softron, Beijing, China). Before the formal experiment, mice were trained for 3 days to become accustomed to the tail-cuff procedure. After baseline recordings, blood pressure was monitored once a week.

### 2.6 Oxidative stress marker detection

After 28 days of treatment, the blood was collected via the orbital vein to prepare serum samples. NRCMs and H9c2 cell lines were seeded in a 6-well plate at 1 × 10^6^ cells/well density and incubated with SU, ASIV, or SU and ASIV. After 24 h, the cell supernatants of each group were collected, and the protein content of the cell supernatants was measured by the BCA protein assay kit (P0012S, Beyotime, China) according to kit instructions. Oxidative stress markers of serum or cell supernatants including LDH, SOD, and MDA were detected by using a commercial kit (Jiancheng Bio, China) according to the manufacturer's protocols.

### 2.7 Mitochondrial superoxide measurement

NRCMs and H9c2 cell lines were seeded in 35 mm culture dishes at a density of 1 × 10^4^ cells/well. After drug treatment for 24h, NRCMs and H9c2 cell lines were incubated with a 5 μmol/L MitoSOX Red indicator (M36008, Invitrogen, USA) for 10 min at 37 °C in the dark. Then, nuclei were counterstained with DAPI (Beyotime, Shanghai, China). Fluorescence images were acquired by using a confocal microscope (ZEISS, Jena, Germany). The fluorescence intensity was measured by Image J (National Institutes of Health, United States). The mean fluorescent intensity of each group was normalized to that of the control group.

### 2.8 Transmission electron microscopy (TEM)

After drug treatment, H9c2 cell lines were trypsinized and precipitated by centrifuge at 1000 rpm for 5 min. Cell precipitates were fixed in 2.5 % glutaraldehyde overnight and then post-fixed in 1 % buffered osmium tetroxide. After dehydration through a graded series of ethanol (50, 70, 90, and 100 %), samples were immersed in acetone and embedding agent and sectioned into 70 nm slices. Ultra-thin sections were imaged by using FEI Tecnai G2 Spirit transmission electron microscope (Thermo Fisher, Holland).

### 2.9 Histopathological assay

After mice sacrificed, the heart was dissected and rinsed 3 times in PBS then fixed in 10 % formalin and embedded in paraffin immediately. Then, sections were prepared and stained with hematoxylin & eosin (H&E) staining (BP0211, Biosci, China) to examine inflammatory infiltrates. The infiltration index in the heart tissues was determined as described previously [26]. Fibrotic lesions were examined by Sirius Red staining (BP094, Biosci, China), and Masson's trichrome staining (BP028, Biosci, China). The fibrosis area was automatically calculated in proportion to the area of the slice by Image J software. The fibrotic lesions of each slide were averaged from 5 fields that were randomly selected.

### 2.10 Immunohistochemical staining

Heart tissue sections were dewaxed in xylene and rehydrated with graded ethanol. Recovery of antigen was performed using 10 mmol/L citric acid buffer (PH 6.0) in a microwave at 100 °C for 10 min. Then, sections were treated with hydrogen peroxidase for 30 min at room temperature to block endogenous peroxidase activity. To suppress background staining, sections were incubated in goat serum for 30 min at room temperature. Sections were incubated with primary antibodies against caspase 3 (1:500, PTG, China) at 4 °C overnight. The next day, the sections were rinsed and incubated with the corresponding secondary antibody for 1 h at room temperature followed by 0.05 % diaminobenzidine (DAB, Sigma, USA) and hematoxylin staining, respectively. Stained tissue sections were scanned with a scanning microscopy imaging system at 200 × magnification and quantitative analysis of immunoreactive cells was performed by Image J software.

### 2.11 TUNEL staining

Dewaxed heart slides, NRCMs and H9c2 cell lines were incubated with 0.3 % Triton-X 100 at room temperature for 5 min. Subsequently, TUNEL-reactions were performed on dewaxed heart sections, NRCMs, and H9c2 cell lines using the one-step TUNEL apoptosis assay kit (Beyotime, Shanghai, China) according to the manufacturer's protocol. Briefly, heart sections were incubated with TUNEL detection reagents labeled with fluorescein isothiocyanate for 1 h at 37 °C in the dark, and apoptotic cells showed green fluorescence. NRCMs and H9c2 cardiomyocytes were incubated with TUNEL detection reagents labeled with Cyanine 3 for 1 h at 37 °C in the dark, and apoptotic cells showed red fluorescence. DAPI was used as a counterstain to highlight nuclei. The samples were visualized under a fluorescence microscope, and the TUNEL^+^/DAPI ratio was quantified by Image J software.

### 2.12 Enzyme-linked sorbent assay (ELISA)

Serum samples from animal experiments were subject to detect cardiac troponin I (cTnI) using commercial ELISA kit (Jiancheng Bio, China) according to the manufacturer's instructions.

### 2.13 Quantitative real-time PCR (qPCR)

Total RNA was extracted with TRIzol reagent (15596018, Thermo Fisher, USA). After assessment of RNA quality and concentration, an equal amount of RNA (1000 ng) was reversely transcribed into cDNA by using the HiScript II Q RT SuperMix (Vazyme, Nanjing, China). Quantitative real‐time PCR was performed in an Opticon Real-Time PCR Detection System (ABI 7500; Life Technology, USA) by using the SYBR Green Premix Pro Taq HS qPCR Kit (AG11718, Accurate Biology, China). GAPDH was used as internal control as indicated. Primers were synthesized by Sangon Biotech (Shanghai, China) and shown in Supplementary Table S1.

### 2.14 Western blotting assay

After indicated treatments, NRCMs, H9c2 cell lines, and heart tissues were lysed and separated by SDS-PAGE, and incubated with primary antibodies at 4 °C overnight after transferring to a PVDF membrane. The primary antibodies used were as follows: anti-COUP-TFII (6434; 1:1000; CST, USA), anti-GAPDH (2118; 1:1000; CST, USA), anti-Cleaved caspase3 (9664; 1:1000; CST, USA), anti-BAX (41162; 1:1000; CST, USA) and anti-Bcl-2 (ab196495; 1:1000; Abcam, UK). Then, the membranes were washed for 10 min with TBST buffer 3 times and incubated with corresponding secondary antibodies (7074S; 1:1000; CST, USA) for 1 h at room temperature. Protein bands were visualized with the enhanced chemiluminescence (ECL) reagent (NCM Biotech, Suzhou China) under the Gel Imaging System (Bio-Rad Co., CA, USA) and quantified by using the Image J software.

### 2.15 Lentivirus infection

GENECHEM (Shanghai, China) created lentiviral particles for COUP-TFII that were both overexpressed and knocked-down, as well as their respective controls. After the lentivirus was prepared, NRCMs and H9c2 cell lines were infected with lentivirus for 12 h, and 10 μg/mL polybrene was also added to increase the infection efficiency. Fresh medium was added to replace the medium containing lentivirus and polybrene 12 h later. The cells were collected 48 h later and the transfection efficiency was detected by using western blotting assays.

### 2.16 Molecular docking

The X-ray crystal structure of the target protein COUP-TFII (PDB ID: 3CJW) was retrieved from RCSB Protein Data Bank operated by the Brookhaven National Laboratory (http://www.rscb.org/pdb). The active site of COUP-TFII was obtained from the literature. Then, the co-crystallized ligand and water molecules were taken out of the complex using Discovery Studio Visualize. Using AutoDock Tools (1.5.6 version), the charges and polar hydrogen were added to the receptor-COUP-TFII. Chemdraw 20.0 was used to create the 3D structure of ligand-ASIV. The ligand-ASIV docking into the active sites of receptor-COUP-TFII was performed by using Autodock 4.2.6, which interprets results in the form of binding energy (Kcal/mol). The best binding pose and molecular interactions of ligand-ASIV with the receptor-COUP-TFII were analyzed by Discovery Studio Visualizer.

### 2.17 Ethics approval and consent to participate

Our animal experimental procedures were performed according to the Guide for the Care and Use of Laboratory Animals of the National Institutes of Health (NIH) of the United States and approved by the Ethics Committee for Experimental Research of Shanghai University of Traditional Chinese Medicine, School of Basic Medical Sciences. Ethics Number: PZSHUTCM201106001.

### 2.18 Statistical analysis

GraphPad Prism 7 software was used to conduct all statistical analyses. Moreover, data were presented as Means ± SD. Normality and homogeneity of variance tests were conducted. The data was of normal distribution and had homogeneity of variance. When there were only two groups, the differences were examined via *t*-test, and when there were more than two groups, they were assessed by one-way analysis of variance (ANOVA).

Retain the following sentence "Differences were considered statistically significant at *P* < 0.05. *P < 0.05, **P < 0.01, ***P < 0.001, ns: no significance

## 3 Results

### 3.1 ASIV attenuated SU-induced hypertension and myocardial injury in mice

**Fig. 1:**
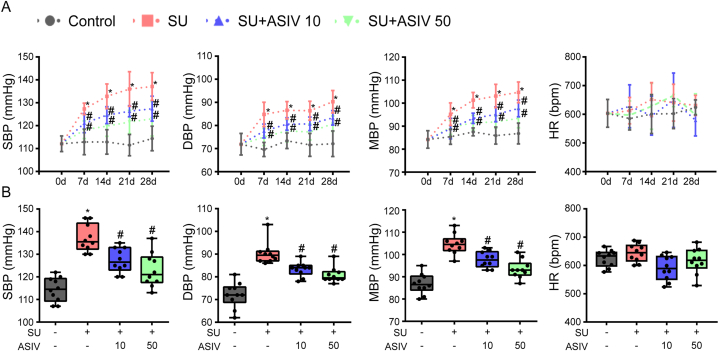
Effects of ASIV on blood pressure in SU-treated mice. (A) Blood pressure (SBP, DBP, and MBP) and heart rate (HR) during the experimental progress among different groups, n = 10. (B) Blood pressure (SBP, DBP, and MBP) and HR at the study endpoint among different groups, n = 10 per group. *p < 0.05 vs. control; ^#^*p* < 0.05 vs. SU.

**Fig. 2:**
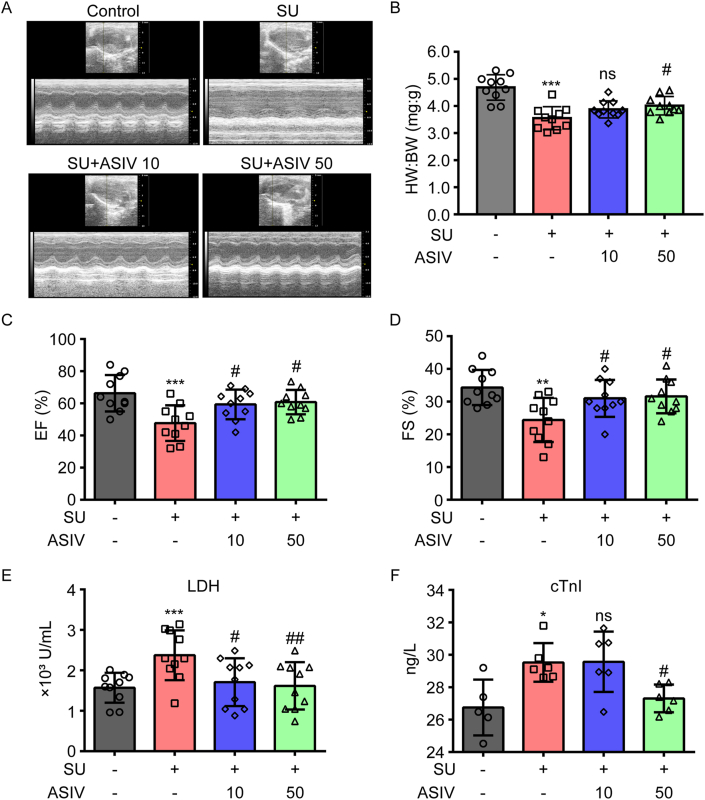
Effects of ASIV on left ventricular systolic function and cardiomyocyte injury markers in SU-treated mice. (A) Representative recordings of M-mode echocardiography in a parasternal long-axis view at the study endpoint. (B) Heart weight to body weight ratio was analyzed. (C–D) Echocardiography measurements of cardiac function (percentage ejection fraction, %EF and fractional shortening, %FS) were conducted, n = 10. (E) Activities of cardiomyocyte injury markers LDH were estimated in mice serum by using diagnostic kits, n = 10. (F) Serum levels of cardiomyocyte injury marker cardiac troponin I (cTnI) were determined using enzyme-linked immunosorbent assay (ELISA) kits, n = 5–6. *p < 0.05, **p < 0.01 and ***p < 0.001 vs. control; ^#^*p* < 0.05 and ^##^*p* < 0.01 vs. SU.

Notably, SU is extensively documented to induce hypertension and myocardial injury in clinical observations [4]. Thus, the cardiovascular parameters of differently treated mice were evaluated to investigate the effects of ASIV on SU-associated cardiotoxicity. SU-treated mice had significantly higher SBP, DBP, and MBP than the control group, and this pressure-increasing effect was alleviated by treatment with 10 and 50 mg/kg ASIV (Fig. 1A and B). However, heart rate was not affected by the different treatments (Fig. 1A and B). To investigate the protective effect of ASIV against SU-induced cardiac dysfunction, we analyzed left ventricle function on day 28 after drug initiation. SU treatment decreased left ventricle EF and FS in mice, which was substantially improved by ASIV, suggesting that SU induces left ventricular systolic and diastolic dysfunction, whereas co-administration of AS-IV could noticeably reverse such adverse events (Fig. 2A–C). As shown in Fig. 2B, the SU-induced decrease in the HW:BW ratio was rescued by ASIV. Moreover, the evaluation of the mice serum samples for cardiac injury biomarkers showed that LDH activities (Fig. 2E) and cTnI levels (Fig. 2F) were significantly higher in the serum of SU-treated mice than in those of the control group. However, animals pretreated with ASIV (10 and 50 mg/kg) showed significantly lower serum LDH activity and cTnI levels than those in SU-treated group did. Collectively, these findings indicated that ASIV ameliorated cardiotoxicity in SU-challenged mice.

### 3.2 Effect of ASIV on SU-induced cardiac histopathological changes

**Fig. 3:**
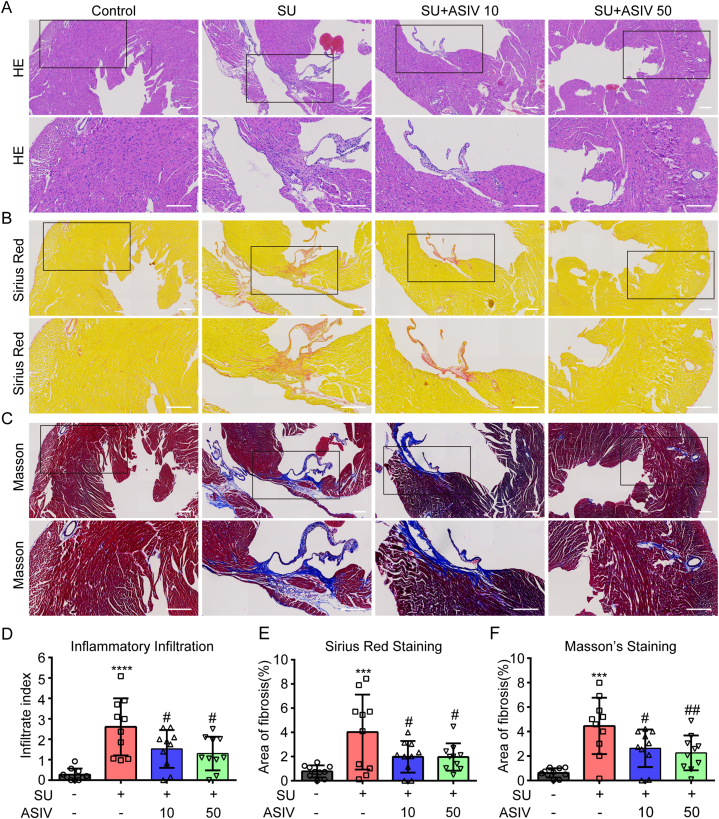
ASIV ameliorated SU-induced cardiac histopathological changes in mice. (A–C) Mouse hearts from in each group were harvested and then hematoxylin and eosin (H&E, inflammation infiltration)-, Sirius Red (fibrotic lesion)- and Masson's Trichrome-stained. Scale bars: 300 μm. (D) Infiltration index of inflammation in each group of mice is shown, n = 9–10. (E–F) Fraction of fibrotic lesion (%) was calculated using Image J software, n = 10. ***p < 0.001 and ****p < 0.0001 vs. control; ^#^*p* < 0.05 and ^##^*p* < 0.01 vs. SU.

We observed that the mice hearts showed abnormal histopathological features after SU treatment, with focally distributed inflammation (hematoxylin and eosin staining) in the perivascular area or the myocardial interstitium (Fig. 3A–C). Furthermore, the infiltration index of SU-treated mice hearts was significantly higher than that of the control hearts. This effect was rescued by ASIV treatment (10 and 50 mg/kg) (Fig. 3D). Moreover, we also observed collagen deposition in heart sections from SU-treated mice using Sirius Red and Masson's trichrome staining, indicating that SU caused cardiac fibrotic proliferation. The area of fibrotic lesions decreased significantly after ASIV treatment (10 and 50 mg/kg) (Fig. 3E–F). These results suggest that ASIV attenuated the SU-induced myocardial histopathological changes.

### 3.3 ASIV suppressed SU-induced myocardial oxidative stress

**Fig. 4:**
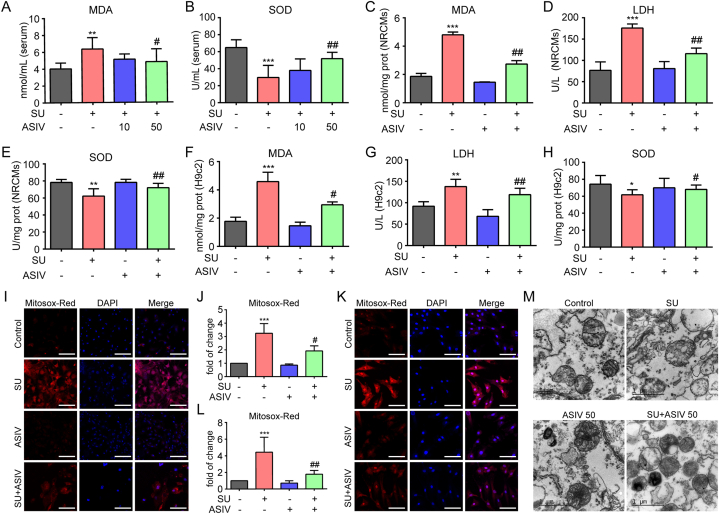
ASIV suppressed SU-induced oxidative stress. (A–B) The content of MDA and SOD activities in mice serum, n = 10. The content of MDA, LDH and SOD activities in NRCMs (C–E), and in H9c2 cell lines (F - H), n = 5. Representative images of Mitosox Red staining and statistical results in NRCMs (I–J), and in H9c2 cell lines (K–L). Scale bars: 150 μm, n = 4. (M) Representative transmission electron microscopy pictures of H9c2 cell lines after treatment. *p < 0.05, **p < 0.01 and ***p < 0.001 vs. control; ^#^*p* < 0.05 and ^##^*p* < 0.01 vs. SU.

We evaluated whether ASIV suppresses SU-induced myocardial oxidative stress in mice hearts, NRCMs and H9c2 cell lines. SU treatment significantly augmented oxidative stress in the heart tissues by inducing higher oxidant MDA levels and lower antioxidant SOD activities than those of the control group (Fig. 4A and B). In contrast, the administration of ASIV (both 10 and 50 mg/kg) significantly ameliorated oxidative stress in SU-treated mice by decreasing the oxidant MDA level and enhancing antioxidant SOD activity (Fig. 4A and B). Before the evaluation of oxidative stress in cell, CCK8 assay was used to select the appropriate drug dosage for the construction of cell model and treatment. It showed that SU induced a dose-dependent viability decrease of H9c2 cell lines, which was reversed by ASIV (Figs. S1A–B). Finally, SU (4 μM) and ASIV (50 μM) were used in all subsequent cell experiments. Moreover, SU treatment significantly augmented oxidative stress in NRCMs and H9c2 cell lines by inducing higher oxidant LDH and MDA levels and lower antioxidant SOD activities than those of the control group (Fig. 4C–H). In contrast, the administration of ASIV significantly ameliorated oxidative stress in SU-treated NRCMs and H9c2 cell lines by decreasing the oxidant MDA and LDH levels and increasing antioxidant SOD activities (Fig. 4C–H). In addition, MitoSox Red indicator, a novel fluorogenic dye for highly selective detection of superoxide in the mitochondria of live cells which was used to directly detect superoxide generated in the mitochondria of NRCMs and H9c2 cell lines. NRCMs and H9c2 cell lines treated with SU showed significantly higher levels of superoxide than the control group did. However, cardiomyocytes pretreated with ASIV showed a significant lower level of superoxide compared with the SU-treated group (Fig. 4I–L). Furthermore, electron microscopy was used to gain insight into mitochondrial structural of H9c2 cell lines. Representative images showed that H9c2 cell lines exposed to SU for 24 h began to exhibit altered mitochondrial morphology, including the appearance of autophagy and the breaking or disappearance of the mitochondrial crest, indicating that SU-induced mitochondrial injury. However, mitochondrial damage caused by SU can be partially improved by the pretreatment with ASIV (Fig. 4M). In all, these results demonstrate that ASIV suppressed SU-induced myocardial oxidative stress both *in vitro* and *in vivo*.

### 3.4 ASIV ameliorated SU-induced cardiomyocyte apoptosis

**Fig. 5:**
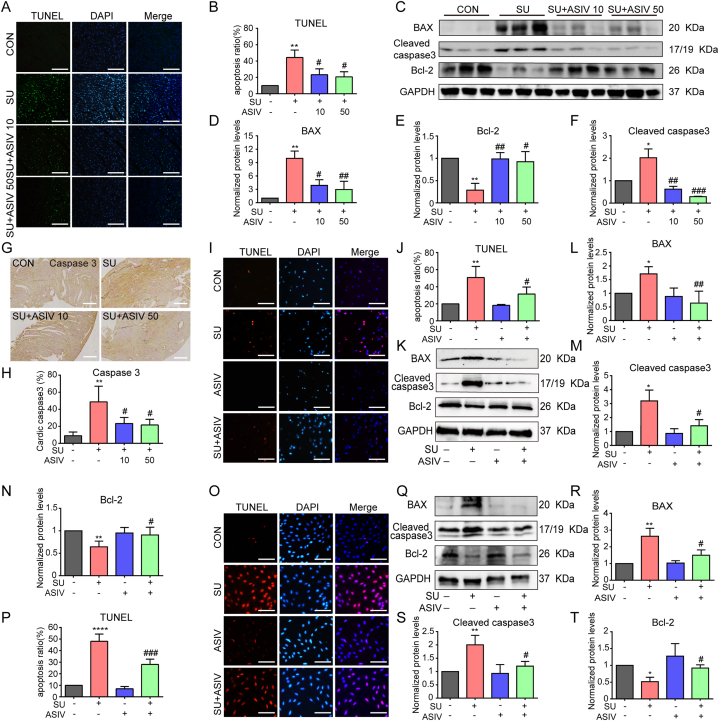
ASIV ameliorated SU-induced cardiomyocyte apoptosis. (A–B) Representative images of TUNEL staining in mice heart tissue and statistical results, scale bars: 300 μm, n = 6. (C - F) Representative Western blot of the BAX, cleaved caspase 3 and Bcl-2 in mice heart tissue and statistical results, n = 3. (G) Mouse heart tissues from each group were immunohistochemically stained for caspase 3, scale bars: 300 μm. (H) Caspase 3 expression level in each group of mice is shown, n = 10. Representative images of TUNEL staining and statistical results in NRCMs (I–J), scale bars: 150 μm, n = 4. Representative Western blot and statistical results of BAX, cleaved caspase 3, as well as Bcl-2 in NRCMs (K - N), n = 4. Representative images of TUNEL staining and statistical results in H9c2 cell lines (O - P), scale bars:150 μm, n = 4. Representative Western blot and statistical results of BAX, cleaved caspase3, as well as Bcl-2 in H9c2 cell lines (Q–T), n = 4. *p < 0.05, **p < 0.01 and ****p < 0.0001 vs. control; ^#^*p* < 0.05, ^##^*p* < 0.01 and ^###^*p* < 0.001 vs. SU.

The cardiomyocyte apoptosis are involved in SU-induced cardiotoxicity [9,11,27]. Therefore, we investigated the effects of ASIV on SU-induced apoptosis in cardiomyocyte. Moreover, TUNEL staining showed that SU treatment increased the number of TUNEL-positive cells in the mice hearts (Fig. 5A and B), NRCMs (Fig. 5I–J) and H9c2 cell lines (Fig. 5O–P), which were significantly decreased by ASIV. Moreover, SU (ranging from 1 to 25 μM) was shown to induce decreased cell viability in a dose-dependent manner in H9c2 cell (Fig. S1A). However, SU (4 μM) co-treatment with ASIV (ranging from 10 to 200 μM) significantly alleviated such cell viability decline (Fig. S1B). Due to the increased TUNEL-positive cells and decreased cardiomyocyte viability induced by SU, changes in the expression of apoptotic markers, such as cleaved caspase-3, Bax, and Bcl-2 were observed in mice, NRCMs, and H9c2 cell lines. Western blotting results showed that the mice (Fig. 5C–F), NRCMs (Fig. 5K–N) and H9c2 cell lines (Fig. 5Q–T) treated with SU showed significantly higher protein levels of the pro-apoptotic factors Bax and cleaved Caspase-3 and lower protein levels of the anti-apoptotic factor Bcl-2 than the control group. However, animals pretreated with ASIV (10 and 50 mg/kg) and NRCMs and H9c2 cell lines pretreated with ASIV (50 μM) showed a significant downregulation of Bax and cleaved Caspase-3 and an upregulation of Bcl-2 compared with the SU-treated group (Fig. 5C–K, Q). Additionally, immunohistochemistry showed increased accumulation of caspase 3 puncta in SU-treated mice hearts, whereas pretreatment with ASIV reduced the accumulation of caspase 3 (Fig. 5G and H). These results suggest that ASIV ameliorates SU-induced myocardial apoptosis.

### 3.5 ASIV restored SU-induced the expression of COUP-TFII

**Fig. 6:**
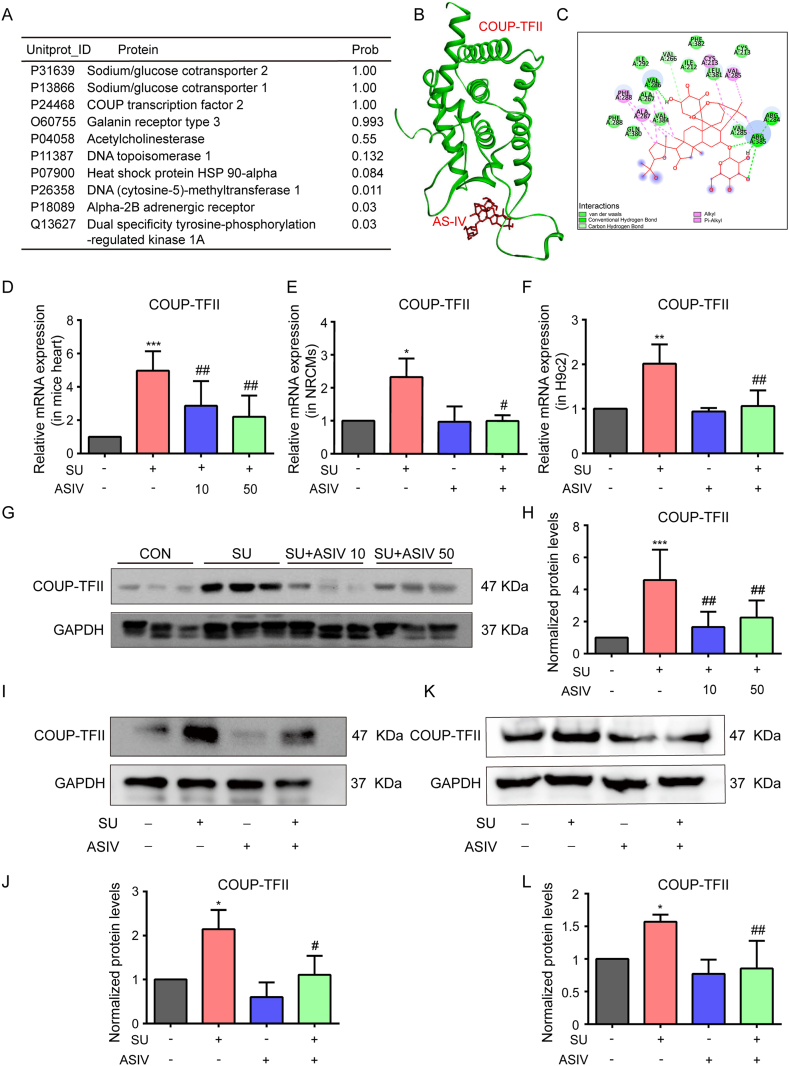
ASIV reversed SU-induced COUP-TFII expression. (A) The main targets of ASIV predicted by TargetNet webserver. (B) Binding mode of ASIV within COUP-TFII. (C) The interactions between ASIV and COUP-TFII. COUP-TFII mRNA levels in mice heart (D), NRCMs (E), and H9c2 cell lines (F) were assessed by real-time qPCR, n = 6. Representative Western blot of COUP-TFII and statistical results in mice heart (G–H), NRCMs (I–J), and H9c2 cell lines (K–L), n = 4. *p < 0.05, **p < 0.01 and ***p < 0.001 vs. control; ^#^*p* < 0.05 and ^##^*p* < 0.01 vs. SU.

To elucidate the molecular mechanism that mediated the cardioprotective effect of ASIV, the potential targets of ASIV were screened through TargetNet webserver (http://targetnet.scbdd.com), an open web server that could be used for netting or predicting the binding of multiple targets for a given molecule [28]. Sodium/glucose cotransporter 2 (SGLT2), Sodium/glucose cotransporter 1 (SGLT1), COUP transcription factor 2 (COUP-TFII) and Galanin receptor type 3 (GALR3) have the highest possibility of interaction with ASIV (Fig. 6A and S1C–D). Molecular docking analysis verified the binding of ASIV to COUP-TFII (Fig. 6B), and the value of the binding energy is −6.9 kcal/mol. It showed that hydrogen bonds exist between ASIV with the amino acids, such as VAL286, ARG284, and ARG385, of COUP-TFII (Fig. 6C). In addition, van der Waals and pi-pi interaction exist in the complex (Fig. 6C), demonstrating that ASIV can strongly bind to COUP-TFII. However, Molecular docking analysis also showed that the interaction between ASIV and SGLT2 was weak (Figs. S1C–D). The effect of ASIV with GALR3 was even weak. Considering that ligands may affect the expression of their target proteins. We further evaluated the effect of ASIV on the expression of its potential targets. Both SU and ASIV did not affect the mRNA expression levels of SGLT2 and GALR3 in H9c2 (Figs. S1E–F) and the expression of SGLT1 was too low to be detected. However, SU significantly induced COUP-TFII mRNA (Fig. 6D–F) and protein (Fig. 6G–L) expression in mice hearts (Fig. 6G and H), NRCMs (Fig. 6I–J) and H9c2 cell lines (Fig. 6K–L), which was reversed by the pretreatment of ASIV. Therefore, we focused on COUP-TFII. These results indicated that COUP-TFII can be regulated by ASIV and SU, and COUP-TFII may be the target of ASIV.

### 3.6 Overexpression of COUP-TFII blocked ASIV-conferred protection against SU-induced oxidative stress and apoptosis in cardiomyocytes

**Fig. 7:**
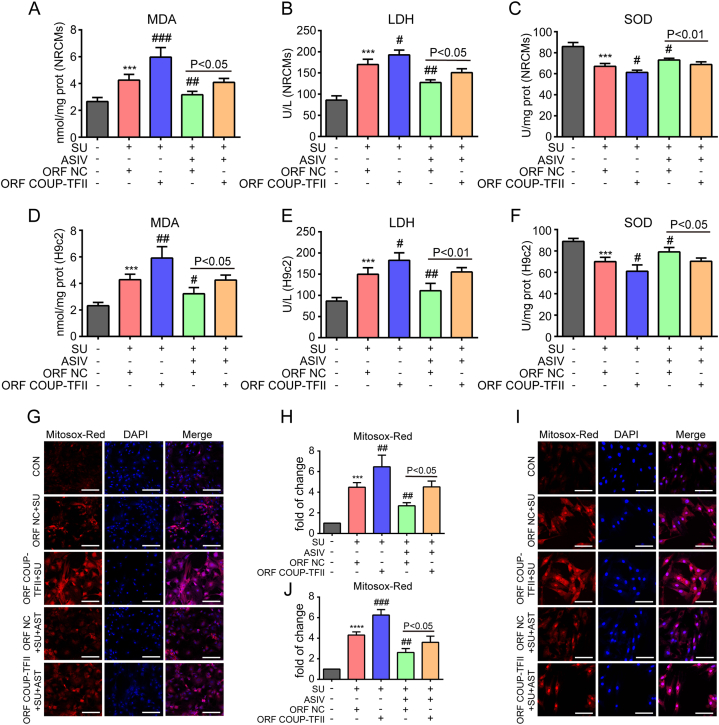
COUP-TFII overexpression blocked the antioxidative effects of ASIV on SU-treated cardiomyocytes. The content of MDA, LDH and SOD activities after COUP-TFII overexpression in NRCMs (A–C), and H9c2 cell lines (D–F), n = 4. Representative images of Mitosox Red staining and statistical results in COUP-TFII-overexpressed NRCMs (G–H), and in COUP-TFII-overexpressed H9c2 cell lines (I–J), scale bars: 150 μm, n = 4. **p < 0.01, ***p < 0.001 and ****p < 0.0001 vs. control; ^#^*p* < 0.05, ^##^*p* < 0.01, ^###^*p* < 0.001 and ^####^*p* < 0.0001 vs. SU.

**Fig. 8:**
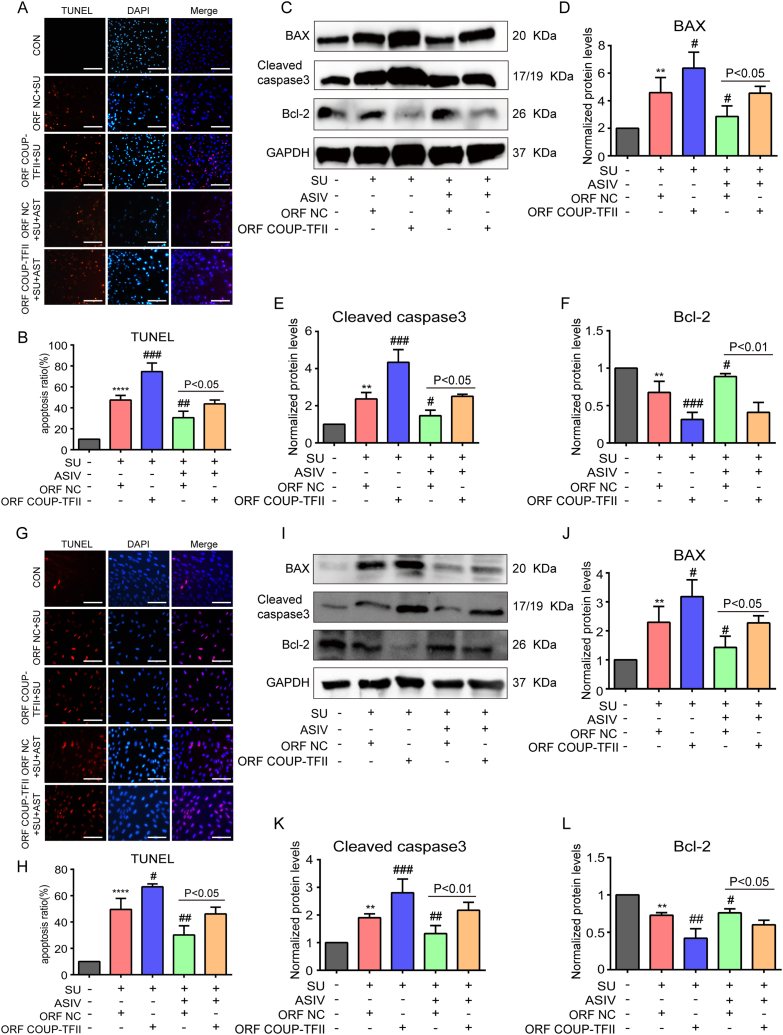
COUP-TFII overexpression blocked the antiapoptotic effects of ASIV on SU-treated cardiomyocytes. (A–B) Representative images of TUNEL staining and statistical results in COUP-TFII-overexpressed NRCMs, scale bars: 150 μm, n = 4. (C - F) Representative Western blot and statistical results of BAX, cleaved caspase 3, and Bcl-2 in COUP-TFII-overexpressed NRCMs, n = 5. (G–H) Representative images of TUNEL staining and statistical results in COUP-TFII-overexpressed H9c2 cell lines, scale bars: 150 μm, n = 4. (I–L) Representative Western blot and statistical results of BAX, cleaved caspase 3 and Bcl-2 in COUP-TFII-overexpressed H9c2 cell lines, n = 5. *p < 0.05, **p < 0.01, ***p < 0.001 and ****p < 0.0001 vs. control; ^#^*p* < 0.05 and ^##^*p* < 0.01 vs. SU.

To establish the role of COUP-TFII in the cardioprotective effects of ASIV against SU-induced cardiotoxicity, COUP-TFII stably overexpressed NRCMs and H9c2 cell lines were constructed via transfecting lentivirus carrying COUP-TFII overexpressing plasmids. Efficient of COUP-TFII overexpression was verified by Western blot in NRCMs (Figs. S2A–B) and H9c2 cell lines (Figs. S2C–D). Next, we examined the oxidative stress and apoptosis in ORF COUP-TFII and ORF NC cardiomyocytes after treated with SU or cotreatment with SU and ASIV. Oxidative stress biomarkers and MitoSox Red assay showed that SU treatment significantly augmented oxidative stress in both ORF COUP-TFII and ORF NC cardiomyocytes by inducing higher oxidant LDH, MDA and superoxide levels and lower antioxidant SOD activity than those of the control group (Fig. 7A–J). Moreover, COUP-TFII overexpression further promoted the induction of oxidative stress by SU by comparing the results of ORF COUP-TFII and ORF NC treated with SU. Notably, COUP-TFII overexpression significantly reduced the antioxidative effect of ASIV by comparing the results of ORF COUP-TFII and ORF NC cotreated with SU and ASIV (Fig. 7A–J). TUNEL staining and Western blot results showed that SU treatment increased the number of TUNEL-positive cells and the expression levels of Bax and cleaved Caspase-3 and lower expression levels of Bcl-2 in both ORF COUP-TFII and ORF NC cardiomyocytes than those of the control group (Fig. 8A–L). COUP-TFII overexpression further augmented the pro-apoptotic effect of SU by comparing the results of ORF COUP-TFII and ORF NC treated with SU, and reduced the anti-apoptotic effect of ASIV by comparing the results of ORF COUP-TFII and ORF NC cardiomyocytes cotreated with SU and ASIV (Fig. 8A–L). Taken together, these results demonstrated that overexpression of COUP-TFII blocked ASIV-conferred protection against SU-induced oxidative stress and apoptosis in cardiomyocytes and ASIV protect against SU-induced cardiotoxicity partially by inhibiting COUP-TFII.

### 3.7 COUP-TFII knockdown alleviated SU-induced oxidative stress and apoptosis in cardiomyocytes

**Fig. 9:**
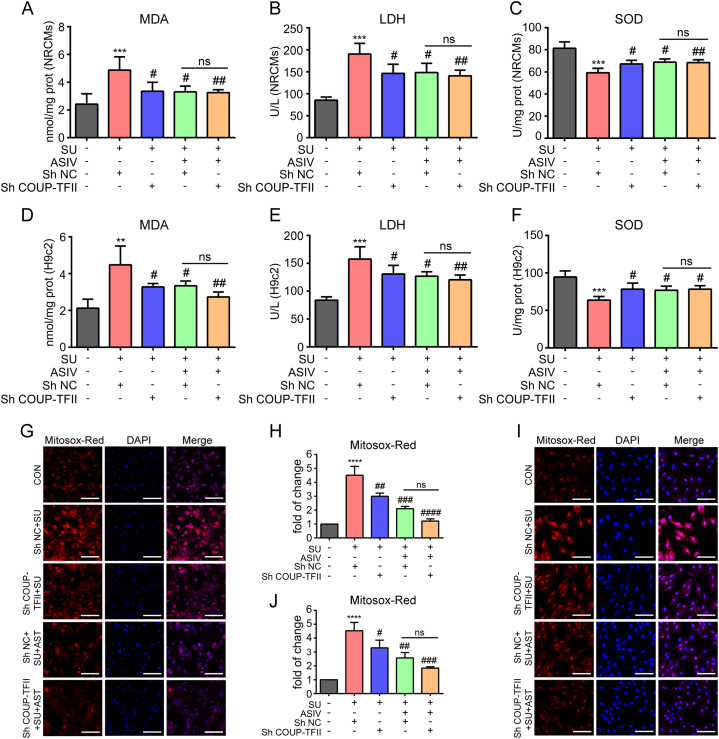
COUP-TFII knockdown suppressed SU-induced cardiomyocyte oxidative stress. The content of MDA, LDH and SOD activities in COUP-TFII knockdown NRCMs (A–C), and H9c2 cell lines (D–F). Representative images of Mitosox Red staining and statistical results in COUP-TFII knockdown NRCMs (G–H), and H9c2 cell lines (I–J), scale bars: 150 μm, n = 4. ***p < 0.001 and ****p < 0.0001 vs. control; ^#^*p* < 0.05 and ^##^*p* < 0.01 vs. SU.

**Fig. 10:**
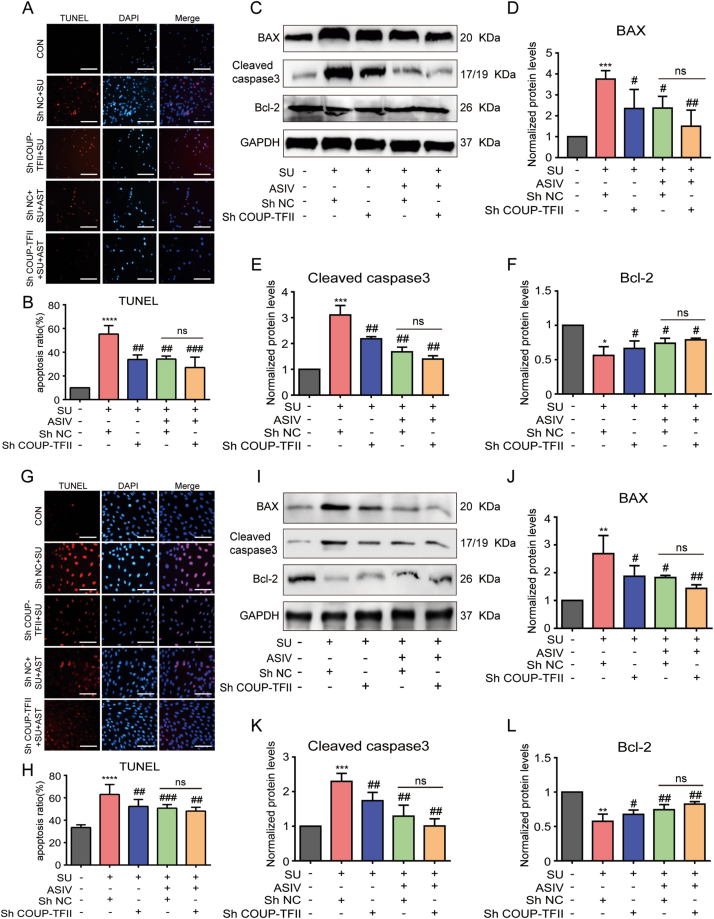
COUP-TFII knockdown suppressed SU-induced cardiomyocyte apoptosis. (A–B) Representative images of TUNEL staining and statistical results in COUP-TFII-overexpressed NRCMs, scale bars: 150 μm, n = 4. (C - F) Representative Western blot and statistical results of BAX, cleaved caspase 3, and Bcl-2 in COUP-TFII-overexpressed NRCMs, n = 5. (G–H) Representative images of TUNEL staining and statistical results in COUP-TFII-overexpressed H9c2 cell lines, scale bars: 150 μm, n = 4. (I–L) Representative Western blot and statistical results of BAX, cleaved caspase 3 and Bcl-2 in COUP-TFII-overexpressed H9c2 cell lines, n = 5. **p < 0.01 and ****p < 0.0001 vs. control; ^#^*p* < 0.05, ^##^*p* < 0.01 and ^###^*p* < 0.001 vs. SU.

To further investigate the role of COUP-TFII in ASIV-conferred protection against SU-induced cardiotoxicity, COUP-TFII stably knockdown NRCMs and H9c2 cell lines were constructed via transfecting lentivirus carrying shRNA against COUP-TFII plasmids. Efficient down-regulation of COUP-TFII expression was verified by Western blot in NRCMs (Figs. S2E–F) and H9c2 cell lines (Figs. S2G–H). Subsequently, we examined the oxidative stress and apoptosis in Sh COUP-TFII and Sh NC cardiomyocytes after treated with SU or cotreatment with SU and ASIV. Oxidative stress biomarkers and MitoSox Red assay showed that SU treatment significantly augmented oxidative stress in Sh NC cardiomyocytes by inducing higher oxidant LDH, MDA and superoxide levels and lower antioxidant SOD activity than those of the control group (Fig. 9A–J). Furthermore, COUP-TFII knockdown significantly reduced SU-induced cardiomyocyte oxidative stress by comparing the results of Sh COUP-TFII and Sh NC cardiomyocytes treated with SU respectively (Fig. 9A–J). However, COUP-TFII knockdown showed similar antioxidative effect of ASIV by comparing the results of Sh COUP-TFII and Sh NC cotreated with SU and ASIV (Fig. 9A–J). TUNEL staining and Western blot results showed that SU treatment increased the number of TUNEL-positive cells and the expression levels of Bax and cleaved Caspase-3 and lower expression levels of Bcl-2 in Sh NC cardiomyocytes than those of the control group (Fig. 10A–L). COUP-TFII knockdown reduced the pro-apoptotic effect of SU by comparing the results of Sh COUP-TFII and Sh NC treated with SU. However, COUP-TFII knockdown showed a tendency to promote the anti-apoptotic effect of ASIV by comparing the results of Sh COUP-TFII and Sh NC cotreated with SU and ASIV respectively, but there was no statistical difference (Fig. 10A–L). Taken together, these results demonstrate that knockdown of COUP-TFII suppressed SU-induced oxidative stress and apoptosis in cardiomyocytes.

## 4 Discussion

In this study, we successfully established a mouse model of SU-associated cardiotoxicity, as evidenced by decreased cardiac function and abnormal histopathological features. Treatment with ASIV ameliorated SU-associated mouse hypertension and cardiac dysfunction, and improved SU-induced mouse heart pathological injury, such as inflammatory infiltration and fibrotic levels. Additionally, ASIV inhibited oxidative stress and apoptosis induced by SU *in vitro* and *in vivo*. Mechanistically, SU-induced cardiotoxicity was mediated via the upregulation of COUP-TFII, which could be markedly improved by ASIV via the inhibition of COUP-TFII. Holistically, we propose that ASIV is an effective means of ameliorating the cardiac side effects of SU.

Clinical practices showed that treatment with SU was linked with serial adverse effects of cardiovascular, including QT prolongation, heart failure and hypertension. Hypertension is the most frequently encountered event of SU-evoked cardiovascular side effect [4,5]. Many studies have been performed to identify potential cardioprotective drugs against SU-induced hypertension, such as amlodipine, macitentan and empagliflozin [29,30]. In our study, we observed the same pressure-increasing effects of SU on mice, including elevated SBP and DBP. Notably, our data showed that ASIV could significantly ameliorated SU-induced mice hypertension, which were supported by previous studies that ASIV could significantly improve l-NAME-induced hypertension and obesity-associated hypertension via modulation of inflammatory reaction [31,32]. Moreover, empagliflozin, a selective inhibitor of SGLT2 by binding directly to SGLT2, has been reported to exert cardioprotective effects in several cardiovascular diseases, including heart failure and diabetic cardiomyopathy [[33], [34], [35]]. Recent study also showed that empagliflozin has a protective role against SU-induced mice hypertension, which may be partially related to its diuretic effect [30]. Because ASIV has a beta-d-xylopyranosyl and a beta-d-glucopyranosyl residues attached at positions O-3 and O-6 respectively, it should be able to bind to SGLT2. Indeed, molecular docking analysis showed a week binding of ASIV to SGLT2 with an evaluated value of 0 kcal/mol of the binding energy, which may be related to the large molecular weight and special three-dimensional structure of ASIV. In addition, our data showed that both ASIV and SU did not affect the transcription level of SGLT2 (Fig. S1E). Furthermore, although the urine volume and urinary sodium of the experimental animals were not recorded in our study, we observed that the sawdust litter in animal cages was basically the same in each group during the experimental progress. Therefore, the antihypertensive effect of ASIV against SU may be related to its anti-inflammatory and antioxidant effects, which may be probably independent of its binding to SGLT2. However, the exact mechanism of the antihypertensive effect of ASIV against SU still needs further investigations.

Cardiomyocyte death is the major pathophysiological cause of cardiovascular disease and cardiotoxicity. We and other groups have verified that SU could significantly increase the death rate of H9c2 cell lines [30,36]. Moreover, we observed significant cardiomyocyte apoptosis and alterations of apoptotic markers both in mice hearts, NRCMs and in H9c2 cell lines. In line with our data, a previous study observed that SU promoted cardiomyocyte apoptosis both *in vivo* and *in vitro* [11]. Studies have verified that maladaptive autophagy promotes the cardiomyocyte apoptosis of SU [11]. In fact, we also observed SU-induced autophagy of H9c2 cell lines by using a transmission electron microscopy detection, which was suppressed by ASIV (data not shown). It has been reported that ASIV could attenuate the myocardial injury caused by adriamycin or lipopolysaccharide by inhibiting autophagy [[37], [38], [39]]. However, it remains elusive the role of autophagy in the antiapoptotic or cardioprotective effect of ASIV on SU-associated cardiotoxicity.

Accumulating evidence shows that up-regulation of COUP-TFII occurs under pathological conditions and aggravates pathological condition, whereas reduction of COUP-TFII expression in these disease models greatly improved pathological conditions [21]. In this study, we observed that the expression level of COUP-TFII was significantly up-regulated in the heart tissue and cardiomyocytes of SU-induced myocardial injury mice and cell models respectively. However, pretreatment with ASIV significantly down-regulated the expression of COUP-TFII. In vitro study showed that knockdown COUP-TFII expression mitigated SU-induced cardiomyocyte oxidative stress and apoptosis, while overexpression of COUP-TFII aggravated SU-induced cardiomyocyte oxidative stress and apoptosis and significantly weakened the antioxidant and anti-apoptotic effects of ASIV. However, whether COUP-TFII is a target of SU and the molecular mechanisms by which ASIV and SU regulate COUP-TFII expression still require further investigation, further experiment still required to evaluate the role of COUP-TFII *in vivo*. At present, COUP-TFII-specific inhibitors are under development and specific COUP-TFII inhibitors have been identified [40,41]. Molecular docking analysis showed that ASIV could bind to the “surface” binding pocket within the human COUP-TFII ligand-binding domain (LBD). Similarly, a previous study reported that 4-methoxynaphthol as an inhibitor of COUP-TFII could bind to the “surface” binding pocket within the human COUP-TFII LBD and decrease COUP-TFII protein levels and activity in hepatoma cells [41]. Nevertheless, no COUP-TFII inhibitor has been applied to clinical practice so far, which may be due to the lack of safety. Moreover, target net and molecular docking analysis are methods to predict the interaction between ASIV and COUP-TFII based on compound and protein structures. However, the interaction between ASIV and COUP-TFII requires further investigation. For example, *in vitro* assays such as surface plasmon resonance (SPR), isothermal titration calorimetry, compound-labeled immunoprecipitation, and fluorescence resonance energy transfer (FRET) can be used. In addition, the direct interaction between ASIV and COUP-TFII can be clarified by crosslinking experiments. Although we have provided concrete evidence that the inhibition of COUP-TFII mediated cardioprotective effects of ASIV on SU-associated cardiotoxicity, it remains elusive whether co-treatment with ASIV would affect the anticancer efficacy of SU. Notably, accumulating evidence indicated that ASIV has antitumor activities in various cancer types [42]. Furthermore, it has been shown that up-regulation of COUP-TFII occurs in a various of cancers and COUP-TFII inhibition showed efficacy for the treatment of cancers [21,[43], [44], [45]]. Therefore, we speculated that ASIV might enhance the anticancer effects of SU through its inhibition of COUP-TFII. However, further investigations are still required to validate the possible enhancing effects of ASIV on the anticancer properties of SU.

## 5 Conclusion

We demonstrated a novel cardiovascular protective effect of ASIV on SU-associated cardiotoxicity. SU-induced cardiotoxicity was mediated via the upregulation of COUP-TFII, which could be markedly improved by ASIV via the inhibition of COUP-TFII. Our data provide novel insights for the treatment of SU-associated cardiotoxicity.

## Institutional review board statement

The animal study protocol was approved by the Ethics Committee of Shanghai University of Traditional Chinese Medicine (ethics number: PZSHUTCM201106001, Approval Data: November 6, 2020).

## Informed consent statement

Not applicable.

## Data availability statement

Data included in article/supp. material/referenced in article.

## CRediT authorship contribution statement

**Wanting Qin:** Writing – original draft, Project administration. **Shaoling Li:** Writing – original draft, Project administration. **Ziji Cheng:** Data curation. **Wenlong Xue:** Writing – review & editing. **Mingyue Tian:** Data curation. **Fangfang Mou:** Data curation. **Haidong Guo:** Writing – review & editing. **Shuijin Shao:** Writing – review & editing. **Baonian Liu:** Writing – review & editing, Writing – original draft, Funding acquisition, Formal analysis, Data curation.

## Declaration of competing interest

No conflict of interest exits in the submission of this manuscript, and manuscript is approved by all authors for publication.

## Figures and Tables

**Figure:**
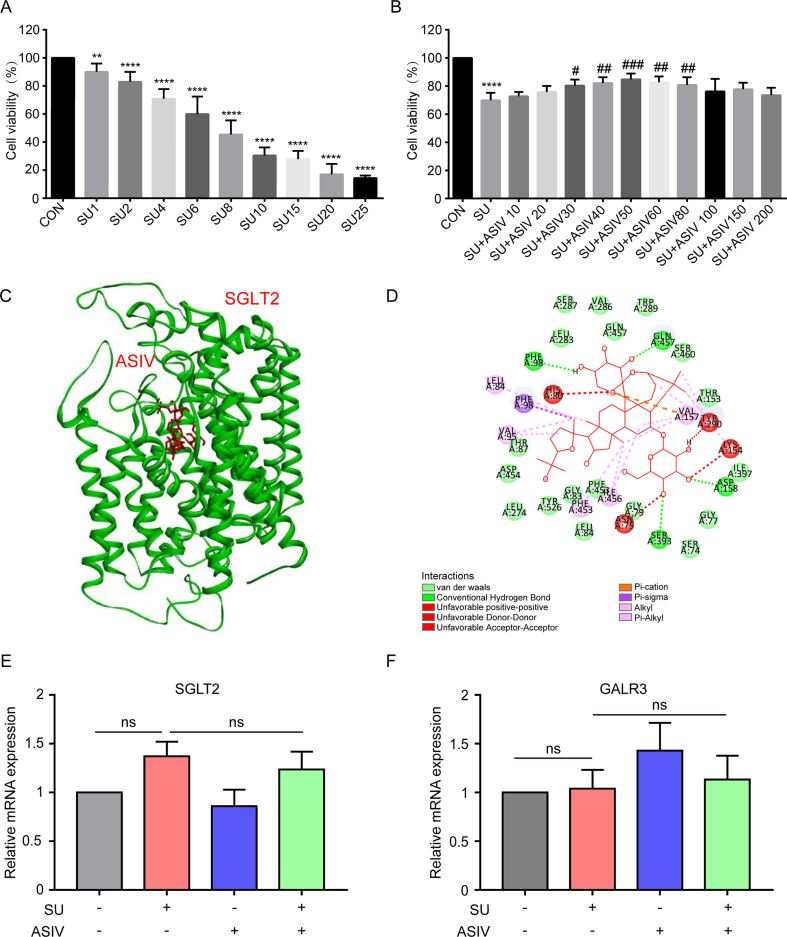
(A) CCK-8 viability assay of H9c2 cell lines treated with vehicle or different concentrations of SU, *n* = 6. (B) CCK-8 viability assay of H9c2 cell lines treated with vehicle (DMSO), SU (4 μM), or SU and ASIV (ranging from 10 to 200 μM), *n* = 6. (C) Binding mode of ASIV within SGLT2. (D) The interactions between ASIV and SGLT2. (E − F) The mRNA expression level of SGLT2 and GALR3 in H9c2 cell lines was analyzed by RT-qPCR. *n* = 4. **p < 0.01 and ****p < 0.0001 vs. control; ^#^*p* < 0.05, ^##^*p* < 0.01 and ^###^*p* < 0.001 vs. SU.

**Figure:**
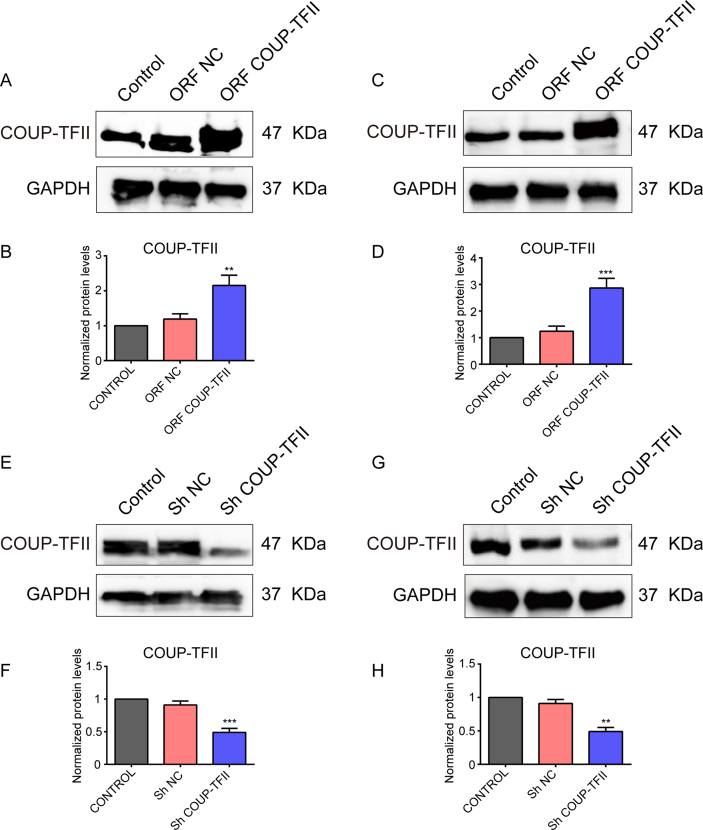
Overexpression or knockdown efficiency of COUP-TFII in NRCMs and H9c2 cell lines. (A - B) NRCMs or (C - D) H9c2 cell lines were infected with ORF COUP-TFII or ORF NC, and COUP-TFII overexpression efficiency was evaluated by western blot, n = 3. (E − F) NRCMs or (G - H) H9c2 cell lines were infected with Sh COUP-TFII or Sh NC, and COUP-TFII knockdown efficiency was evaluated by western blot, n = 3. **p < 0.01 and ***p < 0.001 vs. control.
