# Supplementary material for: Astragaloside IV attenuates sunitinib-associated cardiotoxicity by inhibiting COUP-TFII
Source: Heliyon. 2024 Jan 18;10(3):e24779. doi: 10.1016/j.heliyon.2024.e24779 (PMC10837548; doi:10.1016/j.heliyon.2024.e24779)
Supplement: Multimedia component 1 [file mmc1.docx]

Figure 5C

Full unedited gel for Figure 5C

The lanes in the red box are the lanes in Figure 5C

Antibodies against BAX





Figure 5C

Full unedited gel for Figure 5C

The lanes in the red box are the lanes in Figure 5C

Antibodies against Cleaved caspase3





Figure 5C

Full unedited gel for Figure 5C

The lanes in the red box are the lanes in Figure 5C

Antibodies against Bcl-2





Figure 5C

Full unedited gel for Figure 5C

The lanes in the red box are the lanes in Figure 5C

Antibodies against GAPDH





Figure 5K

Full unedited gel for Figure 5K

The lanes in the red box are the lanes in Figure 5K

Antibodies against Bax





Figure 5K

Full unedited gel for Figure 5K

The lanes in the red box are the lanes in Figure 5K

Antibodies against Cleaved caspase3





Figure 5K

Full unedited gel for Figure 5K

The lanes in the red box are the lanes in Figure 5K

Antibodies against Bcl-2





Figure 5K

Full unedited gel for Figure 5K

The lanes in the red box are the lanes in Figure 5K

Antibodies against GAPDH

**

**

Figure 5Q

Full unedited gel for Figure 5Q

The lanes in the red box are the lanes in Figure 5Q

Antibodies against BAX





Figure 5Q

Full unedited gel for Figure 5Q

The lanes in the red box are the lanes in Figure 5Q

Antibodies against Cleaved caspase3





Figure 5Q

Full unedited gel for Figure 5Q

The lanes in the red box are the lanes in Figure 5Q

Antibodies against Bcl-2





Figure 5Q

Full unedited gel for Figure 5Q

The lanes in the red box are the lanes in Figure 5Q

Antibodies against GAPDH


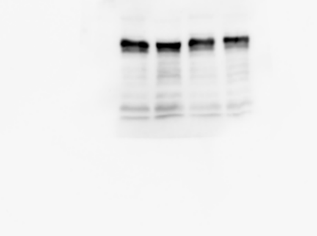


Figure 6G

Full unedited gel for Figure 6G

The lanes in the red box are the lanes in Figure 6G

Antibodies against COUP-TFII





Figure 6G

Full unedited gel for Figure 6G

The lanes in the red box are the lanes in Figure 6G

Antibodies against GAPDH


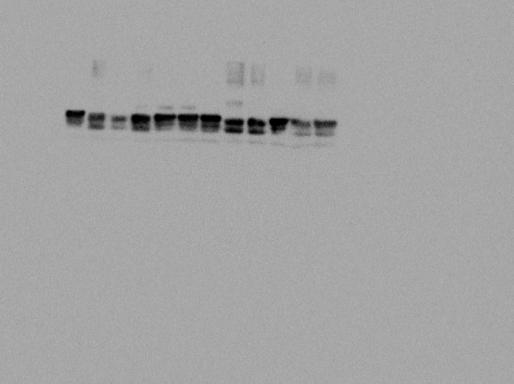


Figure 6I

Full unedited gel for Figure 6I

The lanes in the red box are the lanes in Figure 6I

Antibodies against COUP-TFII





Figure 6I

Full unedited gel for Figure 6I

The lanes in the red box are the lanes in Figure 6I

Antibodies against GAPDH





Figure 6K

Full unedited gel for Figure 6K

The lanes in the red box are the lanes in Figure 6K

Antibodies against COUP-TFII





Figure 6K

Full unedited gel for Figure 6K

The lanes in the red box are the lanes in Figure 6K

Antibodies against GAPDH





Figure 8C

Full unedited gel for Figure 8C

The lanes in the red box are the lanes in Figure 8C

Antibodies against BAX





Figure 8C

Full unedited gel for Figure 8C

The lanes in the red box are the lanes in Figure 8C

Antibodies against Cleaved caspase3





Figure 8C

Full unedited gel for Figure 8C

The lanes in the red box are the lanes in Figure 8C

Antibodies against BCL-2





Figure 8C

Full unedited gel for Figure 8C

The lanes in the red box are the lanes in Figure 8C

Antibodies against GAPDH


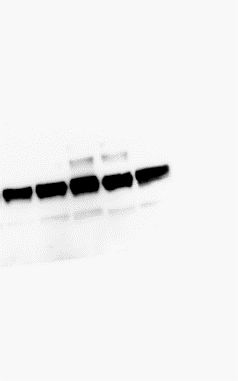


Figure 8I

Full unedited gel for Figure 8I

The lanes in the red box are the lanes in Figure 8I

Antibodies against BAX





Figure 8I

Full unedited gel for Figure 8I

The lanes in the red box are the lanes in Figure 8I

Antibodies against Cleaved caspase3


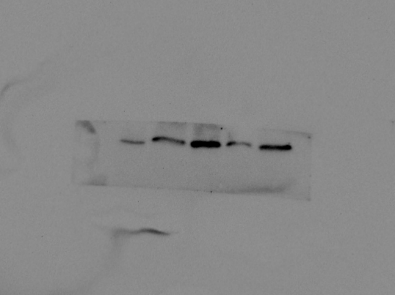


Figure 8I

Full unedited gel for Figure 8I

The lanes in the red box are the lanes in Figure 8I

Antibodies against BCL-2





Figure 8I

Full unedited gel for Figure 8I

The lanes in the red box are the lanes in Figure 8I

Antibodies against GAPDH





Figure 10C

Full unedited gel for Figure 10C

The lanes in the red box are the lanes in Figure 10C

Antibodies against BAX





Figure 10C

Full unedited gel for Figure 10C

The lanes in the red box are the lanes in Figure 10C

Antibodies against Cleaved caspase3





Figure 10C

Full unedited gel for Figure 10C

The lanes in the red box are the lanes in Figure 10C

Antibodies against BCL-2





Figure 10C

Full unedited gel for Figure 10C

The lanes in the red box are the lanes in Figure 10C

Antibodies against GAPDH


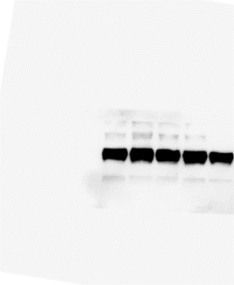


Figure 10I

Full unedited gel for Figure 10I

The lanes in the red box are the lanes in Figure 10I

Antibodies against BAX


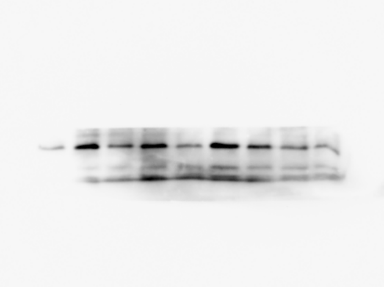


Figure 10I

Full unedited gel for Figure 10I

The lanes in the red box are the lanes in Figure 10I

Antibodies against Cleaved caspase3





Figure 10I

Full unedited gel for Figure 10I

The lanes in the red box are the lanes in Figure 10I

Antibodies against BCL-2





Figure 10I

Full unedited gel for Figure 10I

The lanes in the red box are the lanes in Figure 10I

Antibodies against GAPDH


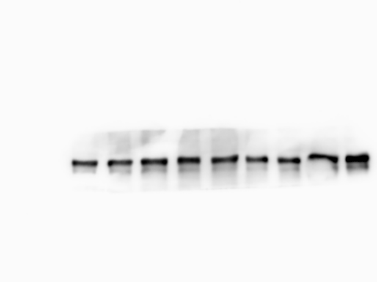


Figure S2A

Full unedited gel for Figure S2A

The lanes in the red box are the lanes in Figure S2A

Antibodies against COUP-TFII





Figure S2A

Full unedited gel for Figure S2A

The lanes in the red box are the lanes in Figure S2A

Antibodies against GAPDH

**
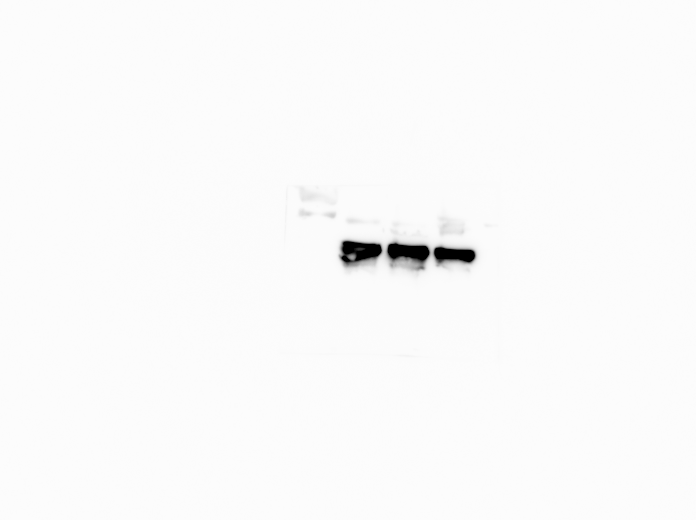
**

Figure S2C

Full unedited gel for Figure S2C

The lanes in the red box are the lanes in Figure S2C

Antibodies against COUP-TFII





Figure S2C

Full unedited gel for Figure S2C

The lanes in the red box are the lanes in Figure S2C

Antibodies against GAPDH





Figure S2E

Full unedited gel for Figure S2E

The lanes in the red box are the lanes in Figure S2E

Antibodies against COUP-TFII





Figure S2E

Full unedited gel for Figure S2E

The lanes in the red box are the lanes in Figure S2E

Antibodies against GAPDH

**
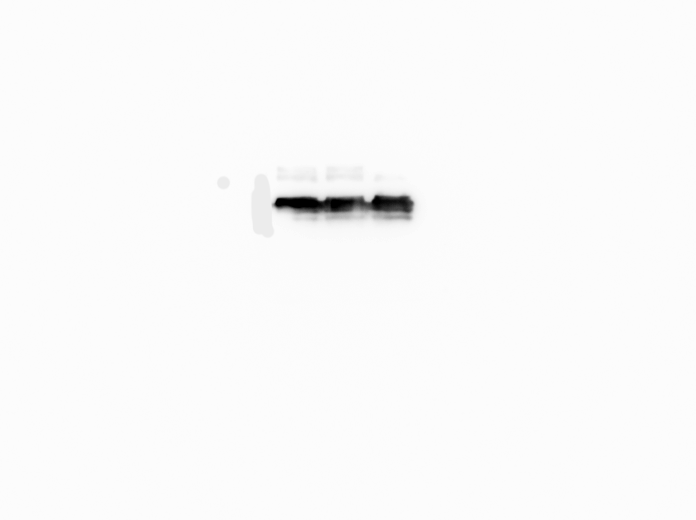
**

Figure S2G

Full unedited gel for Figure S2G

The lanes in the red box are the lanes in Figure S2G

Antibodies against COUP-TFII





Figure S2G

Full unedited gel for Figure S2G

The lanes in the red box are the lanes in Figure S2G

Antibodies against GAPDH
